# Metabolism of parathyroid organoids

**DOI:** 10.3389/fendo.2023.1223312

**Published:** 2023-07-10

**Authors:** Konjeti R. Sekhar, Simona G. Codreanu, Olivia C. Williams, Jeffrey C. Rathmell, W. Kimryn Rathmell, John A. McLean, Stacy D. Sherrod, Naira Baregamian

**Affiliations:** ^1^Division of Surgical Oncology & Endocrine Surgery, Department of Surgery, Vanderbilt University Medical Center, Nashville, TN, United States; ^2^Department of Chemistry and Center for Innovative Technology, Vanderbilt University, Nashville, TN, United States; ^3^Department of Pathology, Microbiology, and Immunology, Vanderbilt University Medical Center, Nashville, TN, United States; ^4^Department of Medicine, Vanderbilt University Medical Center, Nashville, TN, United States

**Keywords:** parathyroid organoids, primary hyperparathyroidism, cryopreserved parathyroid organoids, parathyroid metabolism, untargeted metabolomics, mitochondrial function, glycolytic function, bioenergetic function

## Abstract

**Introduction:**

We successfully developed a broad spectrum of patient-derived endocrine organoids (PDO) from benign and malignant neoplasms of thyroid, parathyroid, and adrenal glands. In this study, we employed functionally intact parathyroid PDOs from benign parathyroid tissues to study primary hyperparathyroidism (PHPT), a common endocrine metabolic disease. As proof of concept, we examined the utility of parathyroid PDOs for bioenergetic and metabolic screening and assessed whether parathyroid PDO metabolism recapitulated matched PHPT tissues.

**Methods:**

Our study methods included a fine-needle aspiration (FNA)-based technique to establish parathyroid PDOs from human PHPT tissues (n=6) in semi-solid culture conditions for organoid formation, growth, and proliferation. Mass spectrometry metabolomic analysis of PHPT tissues and patient-matched PDOs, and live cell bioenergetic profiling of parathyroid PDOs with extracellular flux analyses, were performed. Functional analysis cryopreserved and re-cultured parathyroid PDOs for parathyroid hormone (PTH) secretion was performed using ELISA hormone assays.

**Results and discussion:**

Our findings support both the feasibility of parathyroid PDOs for metabolic and bioenergetic profiling and reinforce metabolic recapitulation of PHPT tissues by patient-matched parathyroid PDOs. Cryopreserved parathyroid PDOs exhibited preserved, rapid, and sustained secretory function after thawing. In conclusion, successful utilization of parathyroid PDOs for metabolic profiling further affirms the feasibility of promising endocrine organoid platforms for future metabolic studies and broader multiplatform and translational applications for therapeutic advancements of parathyroid and other endocrine applications.

## Introduction

Primary hyperparathyroidism (PHPT) is the most common form of parathyroid gland dysfunction, and parathyroid adenomas account for the vast majority of PHPT cases. As the master regulator of calcium metabolism, the parathyroid gland plays a crucial role in overall human metabolism, and hyperfunctioning parathyroid glands alter major metabolic pathways. A comprehensive review of past metabolic studies of PHPT tissues has revealed distinct metabolite differences observed between parathyroid adenomas versus 4-gland hyperplasia, and contrasting metabolic signatures emerged between PHPT and secondary hyperparathyroidism (SHPT) tissues (1). To our knowledge, no prior metabolism studies have been performed using patient-derived parathyroid organoids.

Parathyroid organoids are a novel platform and are actively being explored for both *in vitro* and *in vivo* studies. The Baregamian laboratory has developed a comprehensive organoid program of endocrine patient-derived organoids (PDO) of thyroid, parathyroid, and adrenal neoplasms for translational applications using a fine-needle aspiration (FNA)-based technique (2), and demonstrated preserved multicellular 3-dimensional (3D) ultrastructure, functionally intact, hormone-secreting endocrine organoids of parathyroid, medullary thyroid, and cortisol-secreting adrenal cancer organoids. Parathyroid PDOs revealed a preserved Calcium-sensing receptor (CaSR) expression, autofluorescence characteristic of parathyroid tissues, and sustained PTH secretion over extended periods of time in 3D *in vitro* culture. Other groups have also corroborated some of our findings and confirmed radioisotope uptake by parathyroid organoids derived by a non-FNA method (3).

The metabolism of 3D parathyroid PDOs has not yet been studied and the feasibility of metabolic studies using a 3D *in vitro* platform has not yet been established. This study was designed to expand the multiplatform applications of parathyroid PDOs for metabolic studies and to examine if parathyroid organoids metabolically recapitulate matched PHPT tissues using global untargeted metabolomic profiling. Parathyroid adenomas, the most common subtypes of PHPT, were selected for all metabolic analyses to eliminate inherent metabolite differences between adenomas and 4-gland hyperplasia. Measuring baseline energy metabolism in live parathyroid organoid cells with phenotypical bioenergetic screening of the two major energy-producing pathways, glycolysis and oxidative phosphorylation, will thus help ascertain basal metabolic programming of parathyroid PDOs, and uncover metabolic drivers of parathyroid organoid cell fate, function, and fitness to power future studies.

## Materials and methods

### Experimental workflow

**Figure 1:**
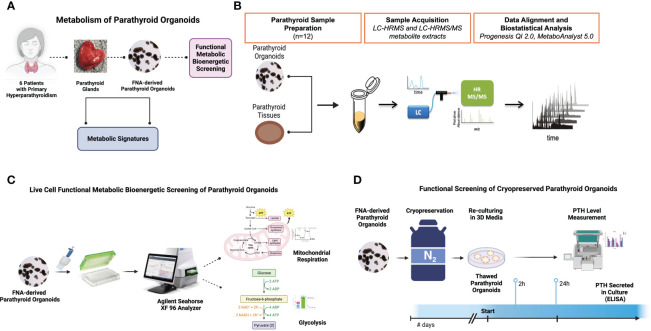
Experimental Design. **(A)** The workflow for the benign parathyroid tissue and tissue matched FNA-derived parathyroid organoid metabolic analyses. **(B)** The workflow for metabolomic analysis of parathyroid organoids and tissues using UHPLC/MS/MS. **(C)** The workflow for bioenergetic studies of organoids using the Agilent Seahorse Analyzer XF platform. **(D)** The workflow for the cryopreserved parathyroid organoid hormone production assessment by ELISA.

The experimental workflow of this study is depicted in **Figure 1**. The parathyroid tissue sample acquisition and generation of parathyroid PDOs **(** **Figure 1A****)** for metabolomic **(** **Figure 1B****)** and bioenergetic flux analyses **(** **Figure 1C****)** were performed. Bioenergetic flux analysis is performed to determine mitochondrial respiration and glycolysis of organoids, followed by preparation of tissues and organoids for metabolomic analysis using UHPLC/MS/MS. This includes identification of metabolites and signaling pathways, and Seahorse flux analysis for mitochondrial respiration and glycolysis rate in parathyroid PDOs. Lastly, the functionality of cryopreserved parathyroid PDOs was determined by their capacity to produce and secrete parathyroid hormone (PTH) within 2-24 hours of thawing and re-culturing (**Figure 1D**). All conceptual workflow figures, including diagrams, were generated using the Biorender illustration online platform (www.biorender.com).

### Human parathyroid tissue collection

With institutional review board (IRB) approval (IRB#222198), we prospectively collected human benign parathyroid tissues from patients (n=6) with primary hyperparathyroidism (PHPT) for the endocrine neoplasia biorepository (ENB). Fresh resected human parathyroid glands were obtained in the operating room at the time of resection for the metabolomic, live-cell functional metabolic bioenergetic, and functional studies post-cryopreservation. Parathyroid tissues collected in the operating room were kept on sterile and chilled surfaces during tissue processing and FNA biopsy and prior to cryopreservation. Tissue collection and cryopreservation was performed under 30 minutes. A small section from each parathyroid tissue was collected (~50-100mg) for metabolomic study, and rapidly cryopreserved by snap-freezing in liquid nitrogen, transported to the lab, and stored at -80^0^C for further analysis.

### Establishing parathyroid patient-derived organoids

Under sterile intraoperative conditions, six parathyroid gland tissues were biopsied *ex vivo* using an FNA-based technique to establish parathyroid patient-derived organoids in semi-solid culture conditions developed, optimized, and previously described by our group (2, 4, 5). Parathyroid PDOs were grown in complete organoid culture media as previously described by our group (2) for 7-10 days, imaged using color brightfield microscopy (Cytation5 BioTek, Winooski, VT), then split into two study arms with the first group of parathyroid PDOs (counted 100 parathyroid PDOs per patient) for metabolomic and bioenergetic analyses, and the second group cryopreserved for downstream functional analysis for PTH secretion.

### Parathyroid organoid sample preparation for metabolomic profiling

Optima grade LC-MS solvents (acetonitrile, methanol, and water) and chemicals (ammonium bicarbonate) for the mass spectrometry metabolomics analyses were obtained from ThermoFisher Scientific (Waltham, MA). Parathyroid and matched parathyroid organoid samples were analyzed via Liquid Chromatography-High Resolution Mass Spectrometry (LC-HRMS and LC-HRMS/MS)-based metabolomics in the Vanderbilt Center for Innovative Technology (CIT) using previously described methods (6–8). The CIT used Quality Control Reference Material (QCRM) and pooled quality control (QC pool) samples that serve numerous purposes, including conditioning of the liquid chromatography column, alignment of retention times, peak-picking, and verification of instrument performance and injection volume reproducibility (9). Parathyroid tissue (n=6) and matched parathyroid organoid samples (n=6, 100 parathyroid PDOs per sample) were lysed in 200 µL ice-cold lysis buffer (1:1:2, acetonitrile: methanol: ammonium bicarbonate 0.1M, pH 8.0) followed by probe tip sonication and normalized via protein amount (35 μg) following a Bicinchoninic acid assay (ThermoFisher Scientific, Waltham, MA). Individual samples were subjected to protein precipitation by addition of 800 µL of ice-cold methanol following the addition of isotopically labeled standards (phenylalanine-D8, biotin-D2 and lauryl carnitine-D3) to determine sample process variability. Precipitated proteins were centrifuged at 10,000 rpm for 10 min after overnight incubation at -80°C. Metabolite extracts were dried *in vacuo* and stored at -80°C. Prior to mass spectrometry analysis, individual extracts were reconstituted in acetonitrile/water (80:20, v/v) containing isotopically labeled standards, tryptophan-D3, and inosine-4N15, and centrifuged for 5 min at 10,000 rpm to remove insoluble material. A pooled quality control sample (QC) was prepared by pooling equal volumes of individual (n=12) samples following reconstitution. The QC sample allowed for column conditioning (eight injections), retention time alignment, and assessment of mass spectrometry instrument reproducibility. The QC sample was injected every four injections.

### Global untargeted metabolomic profiling of parathyroid tissues and patient-matched parathyroid PDOs

Global untargeted mass spectrometry analyses were performed on a high-resolution Q-Exactive HF hybrid quadrupole-Orbitrap mass spectrometer (Thermo Fisher Scientific, Bremen, Germany) equipped with a Vanquish UHPLC binary system (Thermo Fisher Scientific, Bremen, Germany). Parathyroid tissue and matched organoid extracts were separated on an ACQUITY UPLC BEH Amide HILIC 1.7μm, 2.1 × 100 mm column (Waters Corporation, Milford, MA) held at 30°C as previously described (10, 11). Liquid chromatography was performed using a 30 min solvent gradient (30 min) with 5 mM ammonium formate in 90% water, 10% acetonitrile, and 0.1% formic acid (solvent A) and 5 mM ammonium formate in 90% acetonitrile, 10% water, and 0.1% formic acid (solvent B) at 200 µL min^−1^. The injection volume for each sample was 8 μL. Full MS analyses were acquired over the mass-to-charge ratio (*m/z*) range of 70-1,050 in negative ion mode. Full mass scan was acquired at 120,000 resolutions with a scan rate of 3.5 Hz, automatic gain control (AGC) target of 1x10 (6), and maximum ion injection time of 100 ms, and MS/MS spectra were collected at 15,000 resolution, AGC target of 2x10 (5) ions, with a maximum ion injection time of 100 ms.

### Live cell functional metabolic bioenergetic profiling of mitochondrial and glycolytic functions

The Agilent Seahorse microplates provided with the testing kits were coated with Poly-L-lysine solution (P4707, Sigma) and dried. Organoids were added and allowed to bind to the bottom of the wells overnight. The medium was replaced with XF RPMI medium (#103576-100, Agilent). The mitochondrial oxidative phosphorylation was analyzed over time before and after sequential injections. Basal oxygen consumption rate (OCR), ATP-coupled OCR, proton leak, and maximal respiration were quantified by analyzing mitochondrial oxidative phosphorylation before and after injecting the inhibitors: 15 μM Oligomycin, 5 μM carbonyl cyanide 4-trifluoromethoxy phenylhydrazone (FCCP), and 5 μM Antimycin A & Rotenone. The glycolysis stress test was also conducted according to the manufacturer’s instructions. For glycolysis stress test, the medium was replaced with glucose free XF RPMI medium. Basal glycolysis, glycolytic reserve, as well as glycolytic capacity were analyzed by addition of glucose (10 mM), Oligomycin (1 μM), and 2-deoxyglucose (2-DG, 50 mM) sequentially. All samples were run in triplicate and according to the manufacturer’s protocol.

### Metabolomic data alignment and biostatistical and pathway analysis

Progenesis QI v.3.0 (Non-linear Dynamics, Newcastle, UK) was used to review the mass spectroscopy data that was processed and normalized. Pooled QC reference was used to align all the MS and MS/MS sample runs. Unique ions (retention time and m/z pairs) were de-adducted and de-isotoped to generate unique “features” (retention time and m/z pairs). Data was further curated by applying QA practices to the data. Specifically, compounds or metabolites with spectral features with >25% coefficient of variation (CV) in the pooled QC samples were removed. Data were normalized to all features and similarities assessed using the number of features detected in parathyroid tissue (n=6) and matched parathyroid organoids (n=6). Significance was assessed using p-values generated using ANOVA (analysis of variance) from normalized compound abundance data. Metabolites were considered significantly different between the sample groups when p ≤ 0.05 and fold change ≥ |2|.

Accurate mass measurements (< 5 ppm error), isotope distribution similarity, and fragmentation spectrum matching (when applicable) were used to determine both tentative and putative annotations of statically significant compounds. Compounds were searched using METLIN, Human Metabolome

Database (HMDB) (12, 13,) and a highly curated in-house library available in the CIT. Annotations (Level 1-3) (9, 14) were determined for all significant compounds (p ≤ 0.05 and fold change ≥ |2|) with a match to any of the searched libraries or databases. Pathway analyses were performed using MetaboAnalyst 5.0 (www.metaboanalyst.ca) using annotated compounds (statistical significance, p= ≤ 0.05 and fold change ≥ |2|). A pathway impact factor was determined using MetaboAnalyst which reflects the location of an enriched compound within the known canonical KEGG metabolic pathway and its potential impact on the downstream targets. Pathways with p < 0.05 were considered significant.

All samples were normalized to all features. In that, since there is a measurement for every feature ion in all runs (or samples), a ratio can be taken for an individual features ion abundance in a particular run relative to the value in the normalization reference. The software (Progenesis QI) then applies a Log (10) transformation to the calculated ratio to yield a normal distribution on all ratio data within each run for all samples.

### Parathyroid hormone secretion by cryopreserved parathyroid organoids

Cryopreserved parathyroid PDOs from all patients were rapidly thawed and immediately resuspended in warmed complete organoid media in a 50 mL conical tube, as previously described by our group (2, 5). Parathyroid organoids were then centrifuged at 1200 rpm for 5 min at 22^0^C. Supernatant was carefully removed, and the parathyroid organoid pellet was gently resuspended in warmed complete organoid media and plated in a 24-well ultra-low attachment plate (Corning, #3473). Organoids were left in culture for a total of 24 hours. We assessed supernatants for parathyroid hormonal production at 2-hour and 24-hour collection time-points. PTH levels were measured using Enzyme-linked immunosorbent assay (ELISA) kit (Human PTH Kit #ab230931, Abcam) according to the manufacturer’s instructions. All samples were run in triplicate.

### Statistical analysis

Statistical analyses were performed using GraphPad Prism 9 Software (San Diego, CA). Throughout the manuscript, statistical significance is designated as not significant (NS, p > 0.05) and significant (p ≤ 0.05).

## Results

### Metabolomic profiles of parathyroid tissue and organoids

Our group has recently demonstrated the ultrastructural and functional hormonal secretory fidelity of parathyroid PDOs (2); however, it remains unknown whether parathyroid tissue metabolism is recapitulated by cultured parathyroid PDOs, and if any significant metabolic differences exist. To characterize any existing intersystem metabolic differences, we have performed untargeted metabolomic study on parathyroid organoids and matching tissues using Liquid Chromatography-High Resolution Mass Spectrometry (LC-HRMS and LC-HRMS/MS)-based metabolomics. The lysed parathyroid organoids and tissues optimized to equal amounts of protein and isotopic internal standards were added to the samples to assess process variability (**Supplementary Table 1****).**

**Figure 2:**
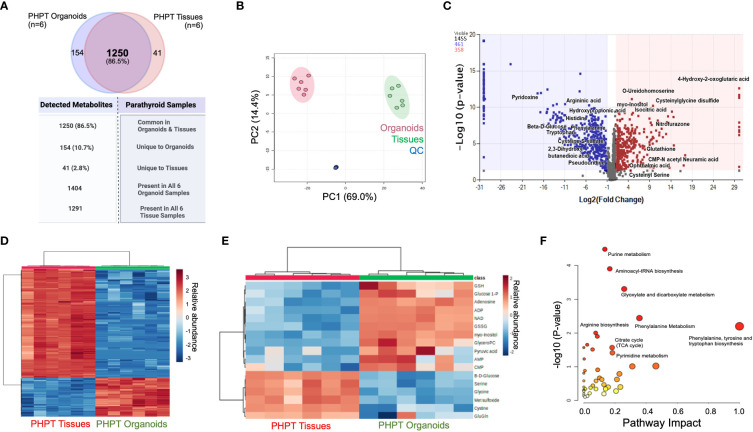
Metabolomic Analysis of Parathyroid PDOs and PHPT tissues. **(A)** The Venn diagram depicts the total metabolites detected and specimen-specific unique metabolites detected. **(B)** The principal component analysis (PCA) of significant metabolites identified in parathyroid PDOs and tissues. Red circles denote organoids, greens circles are tissues, whereas blue circles are quality control (QC) samples (equal composition of both organoids and tissue samples). **(C)** Volcano Plot showing the distribution of metabolic compounds for pairwise comparison based on significance criteria for p-value and fold change. P-values are based on mean difference and variance across the number of sample replicates and are generated using ANOVA. The total number of compounds that met significant criteria for group comparison are colored blue (negative fold change) or red (positive fold change), above the dashed line representing p ≤ 0.05. **(D)** Heat map clustering of the experimental sample groups and compounds. Samples (columns) are clustered by group, and relative feature abundance (rows) across different groups, ranging from low (blue) to high (red) abundance. The heat map was generated for Pareto-scaled, log transformed data using Pearson distance and Average clustering via Metaboanalyst5.0. **(E)** Heat Map clustering of the experimental sample groups and compounds for the most representative compounds for the parathyroid function. Samples (columns) are clustered by group, and relative feature abundance (rows) across different groups, ranging from low (blue) to high (red) abundance. The heat map was generated for Pareto-scaled, log transformed data using Pearson distance and Average clustering via Metaboanalyst 5.0. **(F)** KEGG pathway analysis of annotated metabolites. Analysis using the annotated list of 820 compounds (p ≤ 0.05, fold change ≥ │2│) from parathyroid PDO vs. PHPT tissues. Pathways are mapped by p-values (from pathway enrichment analysis) on the Y-axis, and pathway impact values (from pathway topology analysis) on the X-axis. The node color is based on pathway p-value and the node radius is determined based on pathway impact values.

Comparison of all compounds detected for parathyroid tissue and PDO samples revealed a total of 1455 detected metabolites, with 1404 compounds detected in all PDOs, and 1291 compounds present in all parathyroid tissues. We detected a total of 1250 metabolites **(** **Figure 2A****)** that were common metabolites (86.5%) in both organoid and tissue samples, whereas 154 metabolites (10.7%) were unique to PDOs, and 41 metabolites (2.8%) were tissue-specific. Global principal component analysis (PCA) of organoids and tissue samples was analyzed and plotted **(** **Figure 2B****)** and revealed similar close clustering of tissue samples with PDOs, but widely separated in their clustering.

A total of 820 compounds were annotated as significant using the criteria of p ≤ 0.05 with fold change ≥ |2|. Volcano plot **(** **Figure 2C****)** shows the distribution of compounds of pairwise comparison based on significance criteria of p-value and fold change. A total of 461 compounds are detected with negative fold change (blue dots) and 358 compounds with positive fold change (red dots) when comparing the PHPT tissues to the parathyroid PDOs. A stratified clustering was undertaken using metabolites detected and presented the results in the form of heatmaps **(** **Figures 2D, E****).** Samples (columns) are clustered by group and relative feature abundance (rows) across different groups ranging from low (blue) to high (red) abundance of all compounds.

### KEGG pathway analysis and parathyroid-related metabolites

To understand the differences in signaling pathways in both systems, pathway analysis was performed using the KEGG database on 820 annotated compounds that met the significance criteria (p≤ 0.05 and FC≥|2|, **Figure 2F**; **Supplementary Table 2**). The phenylalanine, tyrosine, and tryptophan biosynthesis pathway emerged with the highest impact factor of 1, followed by glycine, serine, and threonine metabolism and phenylalanine metabolism with impact factors of 0.46 and 0.35, respectively. The highest number of differentially abundant hits or metabolites (n=10) was identified in the purine metabolism pathway (65 total metabolites); however, it did have a lower impact factor of 0.135.

**Figure 3:**
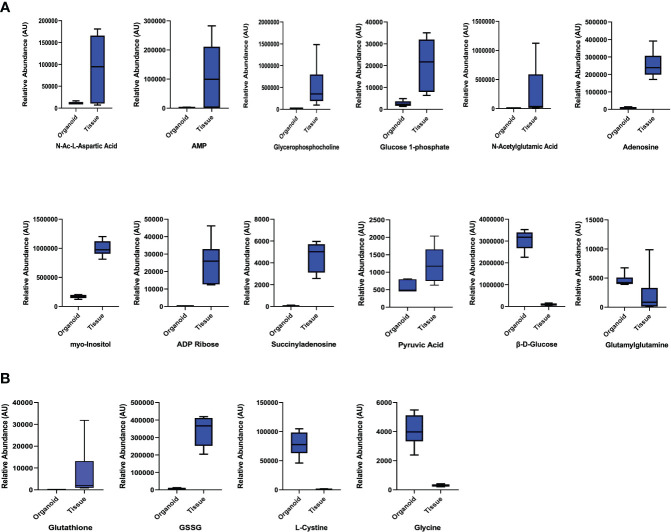
Parathyroid Related Compounds and Glutathione Metabolism. **(A)** Comparison of parathyroid-related compounds in parathyroid PDOs and tissues with significant criteria of p ≤ 0.05 and fold change ≥ |2|. **(B)** Comparison of glutathione metabolites and amino acid precursors of glutathione synthesis.

We also queried data for the parathyroid-related compounds using the significance criteria discussed above. We identified 12 metabolites **(** **Figure 3A**; **Supplementary Table 3**): β-D-glucose and glutamyl glutamine with increased abundance in parathyroid PDOs, 30-fold and 2-fold, respectively, and the remaining 10 metabolites had increased abundance in the tissue samples. The antioxidant profile of parathyroid PDOs and tissues was interrogated next. The glutathione metabolism was significantly altered, where reduced and oxidized glutathione levels were significantly increased in parathyroid tissues, and glutathione synthesis precursors, L-cystine and glycine, were increased in parathyroid PDOs, suggesting that both systems have a differential antioxidant system **(** **Figure 3B****)**. Several metabolites related to amino acids and their analogs were detected with significant differences between parathyroid PDOs and tissues **(** **Supplementary Table 4****)**. Most notably, we observed histidine and cystine increased abundance by more than 100-fold in parathyroid PDOs when compared to the parathyroid tissue samples, and the glutamic acid analog, N-acetyl glutamic acid, increased abundance in parathyroid tissues by 70-fold compared to the parathyroid PDOs.

Altogether, we observed high metabolite fidelity between parathyroid PDOs and their patient matched PHPT tissues, with minimal unique metabolites observed in each of the cohorts.

### Live cell metabolic programming of parathyroid PDOs

**Figure 4:**
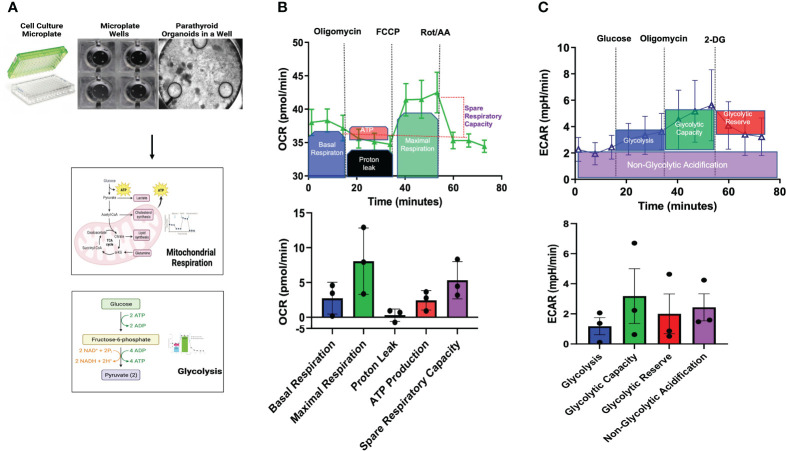
Bioenergetic Flux Analyses. **(A)** Seahorse Agilent culture microplates, an empty well, and a well plated with parathyroid PDOs is imaged by brightfield microscopy to undergo mitochondrial respiration and glycolysis pathway analyses. **(B)** Mitochondrial stress flux analysis results of parathyroid PDOs. Top graph showing the treatment of organoids with modulators of mitochondrial respiration at various time points and description of results. Oxygen consumption rate (OCR) will be measured before and after inhibitor treatment. Bottom bar graph shows the mitochondrial respiration parameters: basal respiration, maximal respiration, proton leak, ATP production, and spare respiratory capacity of parathyroid PDOs. **(C)** Top graph shows extracellular acidification rate (ECAR) after glycolysis stress of parathyroid PDOs. Bottom bar graph shows parameters of glycolysis, glycolytic capacity, glycolytic reserve, and non-glycolytic acidification of parathyroid PDOs.

Metabolic reprogramming has emerged as an indicator of disease susceptibility and developmental abnormalities (15, 16). Metabolomic profiling of parathyroid PDOs and parathyroid tissues reflects a static metabolic snapshot that lacks the insight into the live functional metabolic bioenergetic activity of parathyroid PDOs. These data are the first to demonstrate both the feasibility of utilizing the parathyroid PDOs for extracellular flux analysis by plating organoids in the coated layer of the conventional Seahorse microplate and characterizing the baseline metabolic signature of parathyroid PDOs using the Agilent Seahorse XF technology **(** **Figure 4A****)**. At the cellular level, ATP is the key energy-carrying molecule, and the mitochondrial oxidative phosphorylation and cytosolic glycolysis generate ATP. With the Agilent Seahorse XF96 Analyzer, we measured OCR to assess mitochondrial respiration and extracellular acidification rate (ECAR) associated with the lactate production-linked glycolysis of parathyroid PDOs **(** **Figures 4B, C****)**.

Sequential addition of modulators of mitochondrial function demonstrated increased maximal respiration and high spare respiratory capacity of parathyroid PDOs at baseline. Basal respiration reflects the increased organoid energy demand needed for ATP synthesis. Treatment of parathyroid PDOs with oligomycin, an ATP synthase inhibitor, decreased OCR, which reflects the ATP-linked respiration. The decrease in OCR after oligomycin treatment however was minimal, suggesting that the ATP-linked respiration is not essential for organoids. Proton leak is the remaining basal respiration not coupled to ATP synthesis after oligomycin treatment. The OCR after the injection of the uncoupler agent cyanide 4-(trifluoromethoxy) phenylhydrazone (FCCP) depicts the maximal respiration capacity of the parathyroid PDOs. The difference in maximal respiration and basal respiration denotes the capability of the organoids to respond to energy needs. The OCR after the injection of the Complex I inhibitor rotenone and the complex III inhibitor antimycin A provides information on non-mitochondrial respiration, which is due to cellular enzymes (17, 18). Glycolytic stress assay allowed us to measure the basal glycolysis and glycolytic capacity of the parathyroid PDOs in the setting of ablated mitochondrial ATP production using respiratory modulators. Increased glycolytic activity was measured by ECAR and demonstrated a modest increase in glycolytic capacity following oligomycin administration. This observation suggested that the parathyroid PDOs were operating close to their near-maximal glycolytic capacity, have some glycolytic reserve during metabolic stress, and switch to glycolysis for ATP production. Collectively, the live cell functional metabolic bioenergetic assessment of parathyroid PDOs revealed metabolic energy pathway programming with a robust mitochondrial respiration and ability of these organoids to switch to glycolysis for energy production.

### PTH secretion by parathyroid PDOs post-cryopreservation

**Figure 5:**
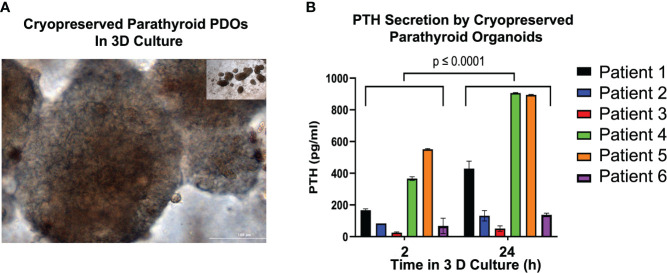
Parathyroid Hormone (PTH) Secretion Analysis of Previously Cryopreserved Parathyroid PDOs. **(A)** Cryopreserved parathyroid PDOs were re-cultured and images captured with bright field microscopy (20x magnification). **(B)** Determination of parathyroid hormone (PTH) in the supernatants of re-cultured, previously cryopreserved parathyroid PDOs collected at 2 and 24 hours after thawing by ELISA. There is significant elevation of PTH levels at 24 hours of culture compared to 2-hour culture (p ≤ 0.0001).

To determine whether cryopreservation negatively impacts the hormonal secretory function of parathyroid PDOs, we re-cultured cryopreserved organoids in media **(** **Figure 5A****)** and collected supernatants for PTH level measurements at 2 and 24 hours after thawing. The microscopic brightfield image of previously cryopreserved and re-cultured organoids **(** **Figure 5A****)** demonstrated areas of cellular budding. We observed consistent PTH secretion by all parathyroid PDOs at the 2-hour time-point post re-culturing, measured by ELISA. Although the PTH levels were variable between patients, a significant increase in secreted PTH levels was achieved by all organoids by the 24-hour time-point **(** **Figure 5B****).**

## Discussion

Three-dimensional parathyroid organoids offer a viable option to study parathyroid-related diseases and serve as a novel and effective modeling tool for *in vitro* and *in vivo* testing. In this study, we successfully utilized 3D parathyroid PDOs for global, untargeted metabolomic and live cell functional metabolic studies as a proof of concept. Parathyroid PDOs accurately recapitulated metabolic profiles of PHPT tissues, thus closely mirroring the complexity of parathyroid tissue metabolic microenvironment. Untargeted metabolomics unveiled significant metabolite fidelity between parathyroid PDOs and their patient-matched PHPT tissues, reflected by the identification of many identical metabolites without significant differences between PDO and tissue cohort metabolites, with only some distinct metabolite signatures emerging as specific to organoids or tissues alone. Parathyroid-specific metabolite differences and metabolic pathway alterations observed in organoids are likely related to metabolic adaptions necessary for organoid formation, proliferation, homeostasis, and parathyroid cell survival under 3D *in vitro* conditions. Similarly, a small fraction (2.8%) of parathyroid tissue-specific metabolites not observed in PDOs may complement the overall recapitulated global metabolic signature of PHPT tissues but may also not be relevant under *in vitro* conditions to parathyroid cell metabolism. At present, these differences remain poorly understood and future studies will help elucidate underlying mechanisms.

PCA analysis of detected metabolites clearly showed distinct separation between organoids and tissue samples, and all organoids and tissues were tightly fit into their own cluster but widely separated from the other, suggesting qualitative fidelity of all samples and emerging metabolic differences. Notably, parathyroid PDOs also appeared to be deficient in converting the surplus of cystine to cysteine and exhibited profound upregulation of histidine and depletion of N-acetyl glutamic acid. The relevance of these findings remains poorly understood, thus additional studies are needed to examine the underlying mechanisms and metabolic consequences of these novel findings.

Successful implementation of parathyroid PDOs for the extracellular flux analyses was achieved with parathyroid organoids plated in the coated layer of the Seahorse microplate within a conventional Agilent Seahorse XF platform. This proof-of-concept approach closely emulated the 3D microenvironment and we assured nearly 95% microwell plating density required for accurate measurements by densely plating various-sized parathyroid PDOs in each well. This method is likely ineffective if individual parathyroid organoid response analysis is desired unless a specialized 3D-printed organoid well is designed to capture a single organoid for precise measurements. With recent development of a 3D Seahorse microplate and new 3D software by the Agilent Seahorse XF platform, individual organoid metabolic phenotype and response measurements will now be feasible and widely accessible to investigators. The 3D extracellular flux analyses can help unlock individual organoid metabolic responses to therapeutic screening and response analysis. We also observed metabolic plasticity in parathyroid PDOs, with oxidative phosphorylation as the predominant energy-producing metabolic pathway at baseline, and Glycolytic switch activated in stress. Future studies focused on exploring metabolic alterations in other parathyroid-related diseases will enable therapeutic response gauging and detection of metabolic plasticity.

Cryopreservation of parathyroid tissues, in the past, has only yielded nearly ~50% tissue viability at the time of autotransplantation, was costly, and has not been widely adopted for tissue biobanking due to these limitations. Parathyroid tissue allotransplantation has also provided limited success, with chemotherapeutic toxicity to parathyroid tissues, which reduced graft viability to nearly 6 months.

Permanent hypoparathyroidism remains a serious clinical issue that significantly impacts patients’ quality of life, is a costly and lifelong condition and in some patients, difficult to manage medically. Parathyroid PDO auto- or allotransplantation could offer a valuable new venue to relieve the debilitating symptoms of hypoparathyroidism.

In this study, we assessed the functional ability of cryopreserved parathyroid organoids to secrete PTH during the initial re-culture period spanning between the first 2-24 hours. These findings support early functional integrity of all cryopreserved parathyroid PDOs and promising probability of improved viability and transplantation of previously cryopreserved parathyroid PDOs with a higher rate of success compared to parathyroid tissue autotransplantation. Future *in vivo* studies will help inform and enable the design of subsequent clinical trials that can alleviate the symptoms of and offer a durable cure for patients with permanent hypoparathyroidism.

In conclusion, parathyroid PDOs were successfully employed for metabolic profiling and further affirmed the feasibility of endocrine patient-derived organoid platforms for future metabolic studies and broader multiplatform and translational applications for therapeutic advancements in parathyroid and other endocrine diseases.

## Author’s note

Presented at the 2023 Symposium on Parathyroid Fluorescence, Geneva, Switzerland.

## Data availability statement

The original contributions presented in the study are included in the article/**Supplementary Material**. Further inquiries can be directed to the corresponding author.

## Ethics statement

The studies involving human participants were reviewed and approved by VUMC IRB. The patients/participants provided their written informed consent to participate in this study.

## Author contributions

KS and NB contributed to the conception of the project; KS, SC, SS, and NB designed the study. Methodology, validation, formal analysis, and investigation was performed by KS, OW, SC, SS, and NB. SC, SS, and JM executed the untargeted metabolomics sample preparation, data acquisition, and data analysis. Resources were provided by JR, WR, JM, and NB. The original Draft was written by KS, SS, SC, OW, and NB and reviewed and edited by KS, SS, SC, JM, OW, JR, WR, and NB. Project funding and supervision was by NB. All authors contributed to the article and approved the submitted version.

## Supplementary material

Supplementary Table 1 Untargeted Metabolomics Quality Metrics. The Center for Innovative Technology (CIT) at Vanderbilt has established and implemented extensive QA/QC practices for untargeted metabolomics studies to ensure robust and unbiased results. The CIT uses Quality Control Reference Material (QCRM) and pooled quality control (QC pool) samples that serve numerous purposes, including conditioning of the liquid chromatography column, alignment of retention times, peak-picking, and verification of instrument performance and injection volume reproducibility. Table shows the metrics obtained for the QC pools used for this project.

Supplementary Table 2 KEGG Pathway Analysis of Annotated Metabolites. Analysis using the annotated list of 820 compounds (p ≤ 0.05, fold change ≥ │2|) from parathyroid PDO vs. PHPT tissues. Only the pathways with a p-value < 0.05 were considered significant. Significant pathways identified were listed in this table with their impact factor.

Supplementary Table 3 Parathyroid Related Compounds. Some of the common metabolites that are identified in parathyroid tissues were analyzed for their differential abundance in the tissues vs organoids. PT-parathyroid tissue; PO-parathyroid organoid.

Supplementary Table 4 Differential Expression of Amino Acids and Analogs. Significant criteria for selection of compounds are p ≤ 0.05 and fold change ≥ |2| except for citrulline.
