# Supplementary material for: Metabolism of parathyroid organoids
Source: Front Endocrinol (Lausanne). 2023 Jul 10;14:1223312. doi: 10.3389/fendo.2023.1223312 (PMC10364603; doi:10.3389/fendo.2023.1223312)
Supplement: Supplementary Table 1 — Untargeted Metabolomics Quality Metrics. The Center for Innovative Technology (CIT) at Vanderbilt has established and implemented extensive QA/QC practices for untargeted metabolomics studies to ensure robust and unbiased results. The CIT uses Quality Control Reference Material (QCRM) and pooled quality control (QC pool) samples that serve numerous purposes, including conditioning of the liquid chromatography column, alignment of retention times, peak-picking, and verification of instrument performance and injection volume reproducibility. Table shows the metrics obtained for the QC pools used for this project. [file DataSheet_1.pdf]

Supplementary Table 1

Measurement	QC Pool Sample Assessment	HILIC neg
		% CV
Process Variability	Biotin – D2	11.43
	Phenylalanine – D8	23.82
	Lauryl carnitine – D3	15.98
Instrument Variability	Tryptophan – D3	1.68
	Inosine – 4N15	0.14

Supplementary Table 2

Pathways	Total	Expect	Hits	Raw p	Impact
Purine metabolism	65	2.18	10	3.28E-05	0.1352
Aminoacyl-tRNA biosynthesis	48	1.61	8	0.000125	0.16667
Glyoxylate and dicarboxylate metabolism	32	1.07	6	0.000494	0.25927
Phenylalanine metabolism	10	0.34	3	0.00362	0.35714
Phenylalanine, tyrosine and tryptophan biosynthesis	4	0.13	2	0.006345	1
Arginine biosynthesis	14	0.47	3	0.009984	0.07614
Alanine, aspartate and glutamate metabolism	28	0.94	4	0.012704	0.08654
Cysteine and methionine metabolism	33	1.11	4	0.022436	0.02184
Citrate cycle (TCA cycle)	20	0.67	3	0.027131	0.1817
Ascorbate and aldarate metabolism	8	0.27	2	0.027162	0
beta-Alanine metabolism	21	0.70	3	0.030917	0.05597
Pyrimidine metabolism	39	1.31	4	0.038904	0.18376
Galactose metabolism	27	0.91	3	0.059078	0.00228
Glycine, serine and threonine metabolism	33	1.11	3	0.095865	0.46284
Histidine metabolism	16	0.54	2	0.098187	0.31147

Supplementary Table 3

Compound ID	Metabolite	Anova (p)	q Value	Max Fold Change	Highest Mean	Lowest Mean
HMDB0000516	Beta-D-Glucose	1.79E-07	1.30E-07	30.751	PO	PT
HMDB0028817	Glutamylglutamine	0.0237401	0.003757	2.146	PO	PT
HMDB0000243	Pyruvic acid	0.0069468	0.001214	2.102	PT	PO
HMDB0000050	Adenosine	3.80E-07	2.44E-07	37.397	PT	PO
HMDB0001138	NAG	0.003444147	0.0006340	70.201	PT	PO
HMDB0000812	NAA	0.032002278	0.0049310	8.1182	PT	PO
HMDB0000912	Succinyladenosine	4.24E-05	1.20E-05	164.489	PT	PO
HMDB0000086	Glycerophosphocholine	7.17E-06	2.82E-06	36.037	PT	PO
HMDB0000211	myo-Inositol	3.85E-09	5.66E-09	5.934	PT	PO
HMDB0000045	AMP	0.0047216	0.00085316	123.323	PT	PO
HMDB0001586	Glucose 1-phosphate	0.0001948	4.58E-05	7.917	PT	PO
HMDB0001178	ADP ribose	3.34E-06	1.54E-06	597.265	PT	PO

Supplementary Table 4

Metabolite	Anova (p)	q Value	Fold Change	Organoids Average ± Std Dev	Tissue Average ± Std Dev
N-Acetylornithine	1.08E-07	8.68E-08	12.40	4086.63 ± 1203.09	329.3492 ± 108.25
Methionine sulfoxide	8.49E-07	4.92E-07	3.43	1986.464 ± 401.70	578.7502 ± 93.50
N-Acetylglutamic acid	0.00344	0.00064	70.20	3877.897 ± 3790. 54	272235.2 ± 445147.2
L-Tryptophan	8.24E-05	2.16E-05	4.81	5408.283 ± 1436 .00	1123.168 ± 843.58
Citrulline	0.117393	0.0169	1.38	4971.899 ± 1475. 61	3584.13 ± 1747.75
N-Acetyl-L-aspartic acid	0.0320	0.00499	8.11	11284.97 ± 2949. 22	91613.69 ± 81300.42
L-Arginine	1.35E-10	3.64E-10	15.63	352814.6 ± 74215.46	22562.2 ± 2749.72
L-Cystine	2.73E-05	8.27E-06	128.41	78577.42 ± 21031.73	611.9112 ± 711.79
L-Serine	2.69E-06	1.3E-06	3.91	31314.52 ± 7230.47	7996.221 ± 1804.73
L-Histidine	1.58E-08	1.89E-08	145.40	64466.53 ± 37166.2	443.3447 ± 139.05
L-Phenylalanine	5.23E-09	7.32E-09	9.07	45068.67 ± 9042.44	4966.138 ± 1014.74
L-Tyrosine	9.65E-07	5.48E-07	3.51	11268.32 ± 2090.98	3207.043 ± 810.46
Glutamylglutamine	0.0237	0.0038	2.14	4609.788 ± 1075.63	2147.602 ± 3808.84
